# Abrogating the Interaction Between p53 and Mortalin (Grp75/HSPA9/mtHsp70) for Cancer Therapy: The Story so far

**DOI:** 10.3389/fcell.2022.879632

**Published:** 2022-04-14

**Authors:** Ahmed Elwakeel

**Affiliations:** Centre for Sport, Exercise, and Life Sciences (CSELS), Coventry University, Coventry, United Kingdom

**Keywords:** mortalin, p53, protein-protein interaction, mortalin-p53 interaction, inhibitors, drug discovery

## Abstract

p53 is a transcription factor that activates the expression of a set of genes that serve as a critical barrier to oncogenesis. Inactivation of p53 is the most common characteristic in sporadic human cancers. Mortalin is a differentially sub-cellularly localized member of the heat shock protein 70 family of chaperones that has essential mitochondrial and extra-mitochondrial functions. Elevated mortalin levels in multiple cancerous tissues and tumor-derived cell lines emphasized its key role in oncogenesis. One of mortalin’s major oncogenic roles is the inactivation of p53. Mortalin binds to p53 sequestering it in the cytoplasm. Hence, p53 cannot freely shuttle to the nucleus to perform its tumor suppressor functions as a transcription factor. This protein-protein interaction was reported to be cancer-specific, hence, a selective druggable target for a rationalistic cancer therapeutic strategy. In this review article, the chronological identification of mortalin-p53 interactions is summarized, the challenges and general strategies for targeting protein-protein interactions are briefly discussed, and information about compounds that have been reported to abrogate mortalin-p53 interaction is provided. Finally, the reasons why the disruption of this druggable interaction has not yet been applied clinically are discussed.

## Introduction

**FIGURE 1:**
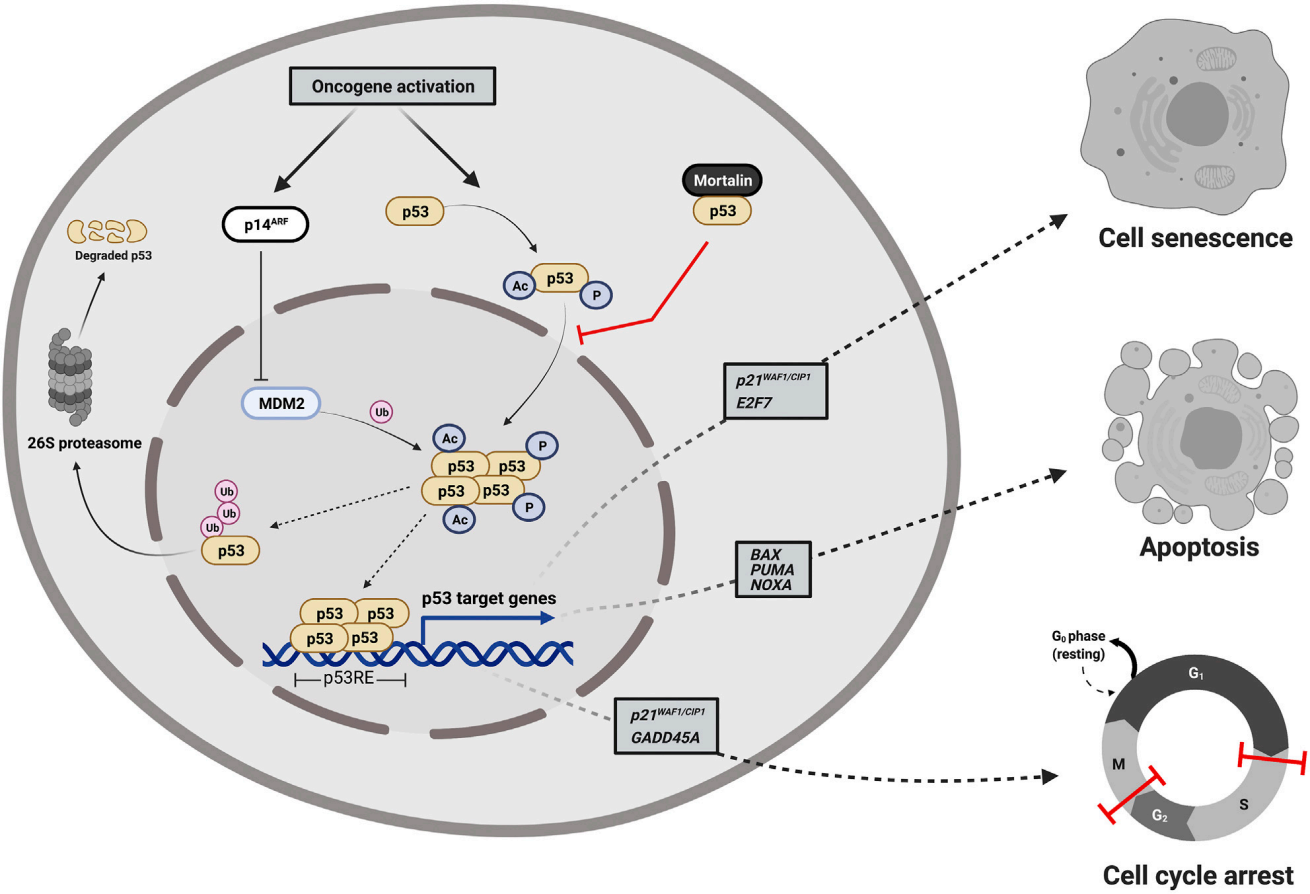
Cellular responses to oncogene-dependent activation of p53 signaling. Oncogenic stress signals activate p53 directly through the activation of various kinases and acetyl transferases that post-translationally phosphorylate and acetylate p53 to be stabilized, accumulated, and translocated to the nucleus and indirectly through the induction of the p14^ARF^ tumor suppressor that binds to MDM2 preventing its interaction with p53 and halting the proteosome-mediated degradation of p53. Upon translocation to the nucleus, tetramerized p53 acts as a DNA-sequence specific transcription factor transactivating a set of genes responsible for tumor suppression programs (apoptosis, cell cycle arrest and cell senescence). Mortalin binds to p53 sequestering it in the cytoplasm and preventing its transcriptional activation functions. The figure was created using (www.app.biorender.com).

**FIGURE 2:**
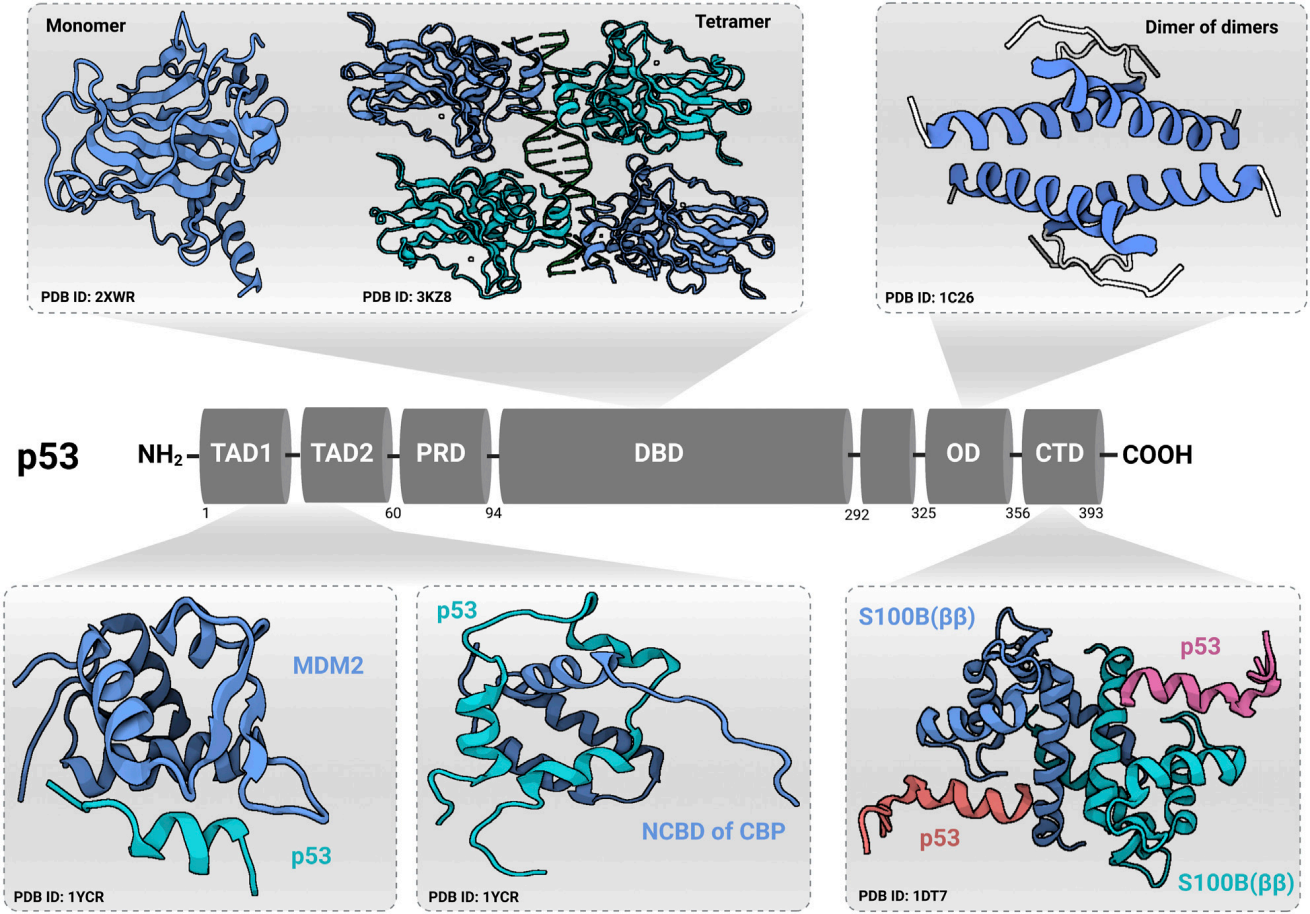
Domain structure of p53. p53 contains an N-terminal transactivation domain (TAD), which can be subdivided into the subdomains TAD1 and TAD2, followed by a conserved proline-rich domain (PRD). The DNA-binding domain (DBD) and tetramerization/oligomerization domains (OD) are connected through a linker region. At the C-terminal end, p53 has a regulatory region called C-terminal domain (CTD). Transactivation domain (TAD) and C-terminal domain (CTD) structures were identified in complex with other regulatory proteins [shown for Mouse double minute 2 homolog (MDM2), nuclear coactivator-binding domain (NCBD) of CREB-binding protein **(**CBP), and S100B (ββ) protein]. Protein Data Bank identification numbers for domain structures are given below each structure. The figure was created (Joerger and Fersht, 2016) using (www.app.biorender.com).

p53 is a sequence-specific DNA binding protein that regulates the transcription of more than 350 confirmed target genes reported from individual gene analyses (Fischer, 2017). Under normal physiological conditions, p53 protein is kept at low levels by means of murine double minute 2 (MDM2), an E3 ubiquitin ligase that directs p53 to degradation by the cellular proteasome machinery. However, as shown in Figure 1, oncogenic signaling activation induces the p14^ARF^ tumor suppressor to bind to MDM2 preventing its interaction with p53 (Weber et al., 1999; Pomerantz et al., 1998) leading to p53 post-translational modification, stabilization, and translocation to the nucleus to transactivate a set of genes responsible for the quite well-understood tumor suppression programs (apoptosis, cell cycle arrest and cell senescence). While p53-mediated apoptosis depends principally on the induction of pro-apoptotic BCL-2 family members (*BAX*, *PUMA*, and *NOXA*), p53 induces cell cycle arrest mainly by the transcriptional activation of *p21* ^*WAF1/CIP1*^ (a cyclin-dependent kinase inhibitor gene) and the growth arrest and DNA damage-inducible-alpha gene (*GADD45A*) (Kastenhuber and Lowe, 2017; Hafner et al., 2019). Furthermore, p53 induces cellular senescence by transactivation of both the *p21* ^*WAF1/CIP1*^ and *E2F7* (the atypical member of the E2F-family of transcription factors) (Rufini et al., 2013). As shown in Figure 2, p53 protein consists of six major domains: (i-ii) two N-terminal transactivation domains (TADs), (iii) a conserved proline-rich domain (PRD), (iv) a central sequence-specific DNA binding domain (DBD), (v) an oligomerization domain (OD), and (vi) a C-terminal domain (CTD) (Boutelle and Attardi, 2021). The fact that p53 is inactivated in more than 50% of all human tumors suggested the indispensability of its role in tumor suppression (Gasco et al., 2002). There are multiple molecular mechanisms behind p53 inactivation. For instance, the mis-sense mutations that occur mainly in its DNA-binding domain result in a loss of its function as a transcription factor and its accumulation in a dysfunctional form in cancer cells. Additionally, the overexpression of p53’s negative regulators (for instance MDM2) is one of the well-established mechanisms that indirectly disables its activity in tumors (Herrero et al., 2016). Furthermore, p14^ARF^ inactivation (mutations, promoter hyper-methylation, or homozygous deletions) has been reported to inactivate p53 in an indirect Mdm2-dependant modality (Gasco et al., 2002).

**FIGURE 3:**
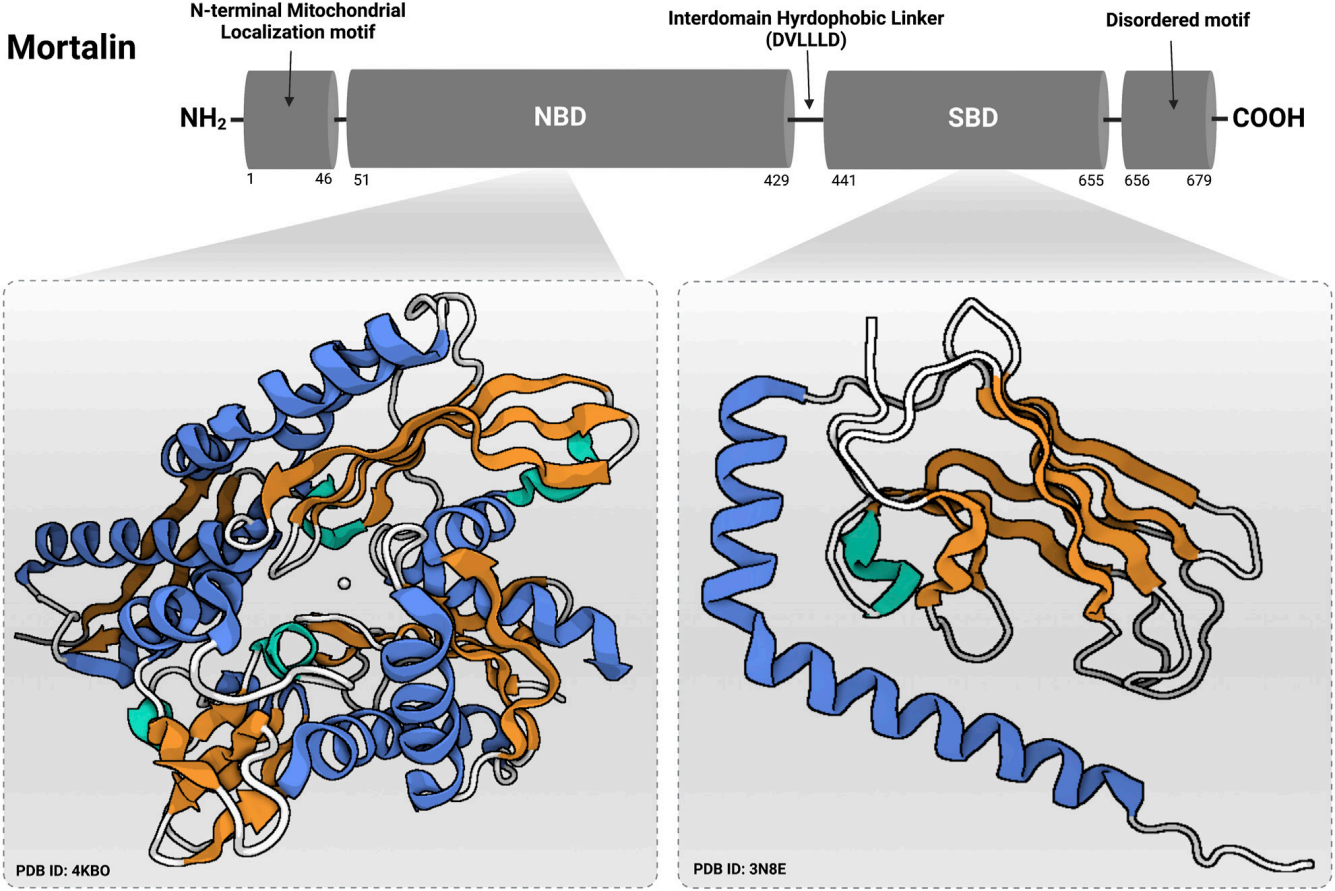
Domain structure of mortalin. Mortalin contains an N-terminal mitochondrial localization motif followed by a nucleotide binding domain (NBD) and a substrate binding domain (SBD). NBD and SBD are connected by a hydrophobic interdomain linker (DVLLLD) (Radons, 2016). At the extreme C-terminal end, mortalin has a disordered motif. Protein Data Bank identification numbers for domain structures are given below each structure. The figure was created using (www.app.biorender.com).

Mortalin is a member of the highly-conserved Hsp70 family of chaperones that was discovered by independent research groups (having multiple births) (Domanico et al., 1993; Wadhwa et al., 1993; Michikawa et al., 1993; Bhattacharyya et al., 1995; Massa et al., 1995), hence, having a multitude of names [for instance, heat shock protein family A (HSP70) member 9 (HSPA9), Peptide-Binding Protein 74 (PBP74), Glucose Regulated Protein 75 (Grp75), C3H Strain Specific Antigen (CSA), and Mitochondrial Heat shock protein 70 (mtHsp70)]. In contrast to murine mortalin which exists in two forms (mot-1 and mot-2) coded by two different genes (originated from two distinct genomic loci assigned to mouse chromosomes 18 and X), human mortalin (hmot-2) is a single 74-kDa protein of a single gene located on chromosome 5, band q31.1 (Kaul et al., 1995; Xie et al., 2000; Wadhwa et al., 1996). Although it can localize to multiple subcellular compartments, the primary location of mortalin is in the mitochondrion. As a member of the conserved Hsp70-family of proteins with essential chaperoning activities that are governed by repeated cycles of binding and release of client proteins under an allosteric control of ATP binding and hydrolysis, mortalin consists mainly of a 42-kD N-terminal nucleotide binding domain (NBD) or ATPase domain and a 25-kD C-terminal substrate binding domain (SBD) or peptide binding domain (PBD)—(shown in Figure 3) (Amick et al., 2014; Radons, 2016). The SBD is divided into a β-sandwich domain (SBDβ) and a 12-kD α-helical lid domain (SBDα). The NBD is divided into four subdomains IA, IB, IIA, and IIB that fold into a pair of lobes to form the nucleotide-binding pocket. The substrate binding site of mortalin has specificity for mixed basic hydrophobic peptide sequences and is contained within the SBDβ. As a ‘‘lid’’ sub-domain, the SBDα covers the peptide binding site in the high substrate-affinity ADP-bound state (a conformation at which the NBD and SBD do not interact with each other but are tethered by the interdomain hydrophobic linker). Upon ADP-ATP exchange, the SBDα undergoes a conformational change leaving the peptide binding site open (client protein release) and returning mortalin to the ATP-apo state (a conformation at which the interdomain hydrophobic linker and the SBDβ dock into the NBD) (Amick et al., 2014). The mechanisms of ATP-dependent allosteric regulation of mortalin’s activity (and other Hsp70-family members) are excellently explained elsewhere (Zuiderweg et al., 2013). Although the crystal structures of both mortalin’s SBD (SBDβ with the first two helices of the SBDα) and NBD were previously solved [PDB IDs: 3N8E (not published) and 4KBO] (Moseng et al., 2019), its full 3D crystal structure has not been resolved yet. Mortalin overexpression was reported in numerous cancerous tissues and tumor-derived cell lines providing ample evidence of its fundamental association with malignancy (Wadhwa et al., 1995; Dundas et al., 2005; Wadhwa et al., 2006; Chen et al., 2014; Jin et al., 2016; Jubran et al., 2017; Sun et al., 2017; Xu et al., 2019). Although the association of mortalin with the intricate process of carcinogenesis is multimodal in nature and includes multiple signaling cascades (Wadhwa and Kaul, 2014; Hu et al., 2016; Xu et al., 2020), it depends mainly on its role as an interaction partner with oncogenic and/or tumor suppressor proteins. For instance, mortalin was identified as a negative regulator of the Raf/MEK/ERK-mediated tumor-suppressive signaling (oncogene-induced senescence) through the physical interaction with MEK1/2. This critical involvement suggested a molecular role in determining the physiological outcomes (proliferation versus growth inhibition) of the aberrant Raf/MEK/ERK signaling pathway (Wu et al., 2013). Other than MEK1/2, and in a different context, mortalin interacts with the human telomerase complex and the heterogeneous nuclear ribonucleoprotein-K (hnRNP-K) in the nucleus causing their stabilization and activation, hence, contributing to malignancy and metastasis (Ryu et al., 2014). Additionally, through the direct binding to C8 and C9 components of the C5b-9 complex, mortalin has been reported to contribute to the removal of the complement membranolytic C5b-9 complex from the K562 human erythroleukemic cell surface, hence, governed the resistance to complement-dependent cytotoxicity (CDC) (Pilzer and Fishelson, 2005; Pilzer et al., 2010; Saar Ray et al., 2014). Finally, in different independent studies, mortalin’s interaction with the p53 tumor suppressor protein was early reported in both mammalian and non-mammalian cell models (Alex Merrick et al., 1996; Wadhwa et al., 1998; Walker et al., 2006). Such an interaction was demonstrated as one of the molecular mechanisms behind the inactivation of the p53’s transcriptional tumor suppression activities (Wadhwa et al., 1998; Kaula et al., 2000).

In this review article, I comprehensively review the chronological identification of mortalin-p53 interaction, cover salient achievements in the identification and development of the compounds that have been reported to possess the potentiality to abrogate such an interaction as possible cancer therapeutic strategy, and finally provide insights into the reasons why the disruption of this notorious protein-protein interaction, although a druggable target, has not been yet translated into clinics.

## Chronological Identification of Mortalin-p53 Interaction

As a member of the Hsp70 family of chaperones with multiple independent discoveries and names (Wadhwa and Kaul, 2014), mortalin’s interaction with p53 was early reported in mammalian cell models in different independent studies. In 1996, in a study in which mortalin was referred to as Glucose Regulated Protein 75 (Grp75) (Alex Merrick et al., 1996), and in an initial attempt to identify individual members of the Hsp70 family that could complex with mutant p53, Bruce Alexander Merrick and colleagues adopted different approaches (immunoprecipitation, immunoblotting and protein sequencing analyses) to firstly report a cytoplasmic interaction between Grp75 and p53. In another study published in 1998, and through the implementation of an immunofluorescence co-localization analyses, Renu Wadhwa and colleagues noticed a co-localization of mortalin (Grp75) and p53 in the murine NIH/3T3 cells (immortalized by the gene overexpression of mot-2 form of murine mortalin), A-172 glioblastoma, U-2 OS osteosarcoma and HeLa cervical carcinoma cells. This co-localization was not observed in normal mouse embryonic fibroblasts (MEF) from CD-1 mice and human mortal TIG-3 fibroblasts. Then, by indirect co-immunoprecipitation analyses on cell lysates of both non-transfected and p53-stably transfected NIH/3T3 cells, they concluded that mortalin not only colocalizes with p53 but also could physically bind to it. Additionally, they reported that this binding had led to a functional inactivation of p53 transcriptional activity possibly through hindrance of its nuclear translocation as follows. Firstly, co-transfection of NIH/3T3 and COS7 cells with an expression plasmid encoding mortalin-2 and the pG13-Luc wild type p53 reporter plasmid resulted in the decline of p53-responsive reporter activity. Of note, such a decline in the wild type-specific p53-responsive reporter activity was reproducible after co-transfection of embryonic fibroblasts from a p53-null (p53^−/−^) mouse with the p53 and mortalin-2 expression plasmids. Secondly, COS7 cells that were transfected with an expression plasmid encoding mortalin-2 showed a lower level of two p53 target genes (*MDM2* and *p21* ^*WAF1/CIP1*^). Thirdly, the wild type p53 was hindered from shuttling to the nucleus in NIH/3T3 cells transfected with a plasmid encoding GFP-tagged mortalin-2 protein after 48-h of serum starvation as a source of stress that should have governed the routine p53 signaling activation (Wadhwa et al., 1998). The authors in this study provided evidence for a physical protein-protein interaction that led to a functional inactivation of the p53 transcriptional programs. Later, in the year 2000, mortalin-p53 interaction was confirmed by another research group (Marchenko et al., 2000). Mortalin-p53 interaction was reported in both mammalian and non-mammalian cancer models (Walker et al., 2006). In this report, the authors showed by co-immunoprecipitation analyses that mortalin could bind to p53 in the cytoplasm of leukemic clam hemocytes; an interaction that was not found in normal hemocytes.

In several independent studies, both the mortalin binding site of p53 and the p53 binding site of mortalin were mapped. Firstly, the mortalin binding site of p53 was found to be localized to the C-terminal 312–352 amino acid (aa) residues within its tetramerization/oligomerization domain (Wadhwa et al., 2002). This finding was further confirmed using p53 C-terminal peptides (or mortalin-binding p53 fragments) that competed with the endogenous p53 and resulted in its stabilization and translocation to the nucleus (Kaul et al., 2005). Then, other studies conducted by different research groups suggested that, not only within its tetramerization/oligomerization domain, mortalin could also bind to p53 within its C-terminal domain (361–393 amino acid residues) (Iosefson and Azem, 2010; Gabizon et al., 2012). Of note, it was reported that the tetrameric structure of p53 is not required for its association with mortalin (Iosefson and Azem, 2010). Secondly, based on results that had been obtained using cell lysates, the p53 binding domain of mortalin (both mot-1 and mot-2 forms) was mapped to its N-terminal amino acid residues 253–282 within the ATPase domain (Kaul et al., 2001). Then, in a screening step of an array of peptides that could bind to p53 (Gabizon et al., 2012), the ATPase domain (specifically the 266–280 amino acid residues) of mortalin was confirmed as a p53 binding domain. Conversely, after the heterologous expression of both the ATPase domain (51–436 amino acid residues) and the peptide binding domain (PBD) (434–679 amino acid residues) of mortalin followed by pull-down analyses, it has been demonstrated by another research group that mortalin’s peptide binding domain (PBD)—but not the ATPase domain–associates with p53 (Iosefson and Azem, 2010). This controversy was justified as possible modification or additional interacting proteins could affect the nature of the interaction between p53 and mortalin (Iosefson and Azem, 2010). For instance, as the p53 binding domain of mortalin (253–282 amino acid residues) includes a potential interaction motif for the mortalin’s J-domain co-chaperone Tid1 (Ahmad et al., 2011), Joseph Amick and colleagues have suggested that Tid1 could possibly pass p53 onto the mortalin’s PBD while itself docking onto the ATPase domain as the interaction between mortalin and p53 has been shown to be regulated by Tid1 (another interaction partner of p53) (Ahn et al., 2010; Trinh et al., 2010; Amick et al., 2014).

## Challenges and Strategies for Targeting Protein-Protein Interactions

Compared to the classical model of targeting protein–ligand interaction, for instance, enzyme-substrate modulation and receptor-ligand modulation, for multiple reasons, targeting protein-protein interaction is considered a challenging approach in drug discovery and development (Lu et al., 2020). Firstly, compared with enzymes or receptors, there are no endogenous small molecular substrates or ligands to be mimicked while designing an abrogator for protein-protein interaction. Secondly, the interface of protein-protein interaction is large, flat, and hydrophobic with few grooves or pockets with which an inhibitor could bind (Buchwald, 2010). Accordingly, an effective protein-protein interaction abrogator should cover a large surface area and establish many hydrophobic interactions which are challenging criteria from a pharmacokinetic perspective. Thirdly, the amino acid residues involved in the interface of protein-protein interaction are either continuous and/or discontinuous in their respective protein structures. Hence, the interacting proteins are bound with a too high affinity to be inhibited by small molecules (Ivanov et al., 2013) and it is difficult to recruit short peptide chains derived from the protein structure as starting points to be mimicked for designing peptidomimetic drugs (Mabonga and Kappo, 2019). Owing to the aforementioned challenges, protein-protein interactions had been previously considered “undruggable” because the classic medicinal chemistry methodologies for designing and/or identifying protein-protein interaction modulators are used to be less effective. However, over the past few decades, considerable theoretical and technological progress has played a key role in developing different strategies to identify hits and leads to target pathological protein-protein interactions. For instance, the emergence of “hot-spots”—a small subset of amino acid residues that contributes to most of the binding free energy of protein–protein interactions—overcomes the problem that a protein-protein interaction modulator should cover a large surface area at the interfaces. In the following section, modern strategies to identify protein—protein interaction modulators will be briefly summarized. Basically, three major strategies could be implemented: (i) screening strategies including high-throughput screening (HTS) and virtual screening, (ii) designing strategies (or structure-based drug design) including hot spot-based design and peptidomimetic design, and finally (iii) screening-then-designing strategy called fragment-based drug discovery (FBDD) (Sheng et al., 2015; Lu et al., 2020).

### High-Throughput Screening

High-throughput screening (HTS) is an automated method performed in microtiter plates in 96-, 384-, or 1536-well formats to test thousands of compounds to identify ‘‘hits’’ with the potential to abrogate the interaction between two proteins. As a classical and well-established method for discovering hits against conventional drug targets, for instance, enzymes and receptors, the compound libraries historically collected or designed for high-throughput screening (HTS) are not suitable to identify hits as protein-protein interaction abrogators due to the unique nature of the interfaces between any two interacting proteins. However, efforts are currently being exerted by pharmaceutical companies to have a broad compound library to possess the required chemical diversity that could meet protein-protein interaction as a drug target (Mullard, 2012). Furthermore, construction of innovative and biologically relevant libraries with improved diversity and complexity has emerged; for instance, the highly efficient synthetic chemistry-based compound libraries [multi-component reactions (MCRs), diversity-oriented synthesis (DOS), biology-oriented synthesis (BIOS), and Cascade-inspired reactions] (Zhuang and Sheng, 2018). As there is no enzymatic readout associated with the binding of two proteins, the selection of a suitable assay and assay read-out is a critical factor in the success of HTS. Previously, several assays have been widely used to identify protein-protein interaction inhibitors, for instance, Fluorescence Polarization (FP) assay (Keap1/Nrf2 interaction inhibitor) (Hu et al., 2013), Time-Resolved Fluorescence Resonance Energy Transfer (TR-FRET) assay (14-3-3/Bad interaction inhibitor) (Du et al., 2013), Bioluminescence Resonance Energy Transfer (BRET) assay [Plk1 Polo-Box Domain (PBD) interaction inhibitor] (Normandin et al., 2016), Amplified luminescent proximity homogeneous assay Screen (AlphaScreen) [KRAS/PDEδ interaction inhibitor] (Zimmermann et al., 2013), and Cell-based Bioimage Redistribution [mortalin/p53 interaction inhibitor] (Gao et al., 2012; Mabonga and Kappo, 2019; Putri et al., 2019; Elwakeel et al., 2021; Sari et al., 2021).

### Virtual Screening

Virtual screening is a computer-aided approach which emerged as a complementary technique to support high-throughput screening (HTS) in the discovery and development of protein-protein interaction modulators. It is defined as the professional application of specialized computer software to screen out hits from virtual compound libraries. Virtual screenings reduce the number of compounds to be screened in the actual high-throughput bioassays, hence, the time and cost can be significantly decreased. Virtual screening strategies are classified into ligand-based virtual screening (LBVS) and structure-based virtual screening (SBVS). Different methods of virtual screening that could be applied in the discovery and development of protein-protein interaction inhibitors were excellently reviewed elsewhere (Macalino et al., 2018; Wu et al., 2019). Virtual screenings were previously applied in the successful identification of protein-protein interaction inhibitors, for instance, TCF/β-catenin (Tian et al., 2012), 14-3-3/aminopeptidase N (Thiel et al., 2013), Ubc13/Uevl (Scheper et al., 2010), mortalin/p53 (Pham et al., 2021; Utomo et al., 2012; Nagpal et al., 2017; Hartati and Djauhari, 2020), and MDM2/p53 (Lawrence et al., 2009).

### Fragment-Based Drug Discovery

Fragment-based drug discovery (FBDD) is a promising strategy for generating a lead compound with the potentiality to abrogate the interaction between two proteins. Firstly, it starts with a screening step to identify small chemical fragments or moieties (∼200 Da), which may only bind at a low millimolar affinity range to their target protein (at or near the interface with a partner interacting protein). The positively identified chemical fragments are then subjected to an “evolution” process by the expansion or the linkage to other small chemical moieties that bind to nearby regions on the same target protein to design a “lead” with stronger affinity. These “leads” are then subjected to an extensive optimization process via medicinal chemistry approaches and may then be entered into preclinical studies for validation (Mabonga and Kappo, 2019). As the screened small chemical fragments are intrinsically weak binders, the methods used for screening have to be more sensitive than those used in their high-throughput screening counterparts, for instance, Disulfide trapping (Tethering), Protein-observed NMR, Ligand-observed NMR, Surface plasmon resonance (SPR), Mass spectrometry (MS) and others (Magee, 2015). FBDD is considered a better approach than HTS for the identification of protein-protein interaction abrogators because the reduced complexity and the enhanced chemical space coverage of the screened small chemical fragments increase the probability of finding binders to target proteins on the interfaces of the interaction with their partners. FBDD has been proven useful in the successful identification of protein-protein interaction inhibitors, for instance, inhibition of the Bcl-xL interaction with BH3 peptides of proapoptotic Bcl-2 family members (Bak and Bad) (Petros et al., 2006), Ras interaction with SOS (Maurer et al., 2012), the interaction of RPA70N [70 kDa subunit of the Replication protein A (RPA) trimer] with ATRIP [ATR-interacting protein] (Waterson et al., 2013), the interaction of Myeloid Leukemia 1 (Mcl-1) with BH3-containg peptides (Friberg et al., 2014; Petros et al., 2014), and the interaction of bromodomain adjacent to zinc finger domain protein 2B (BAZ2B) with the histone H3 peptide acetylated at K14 (H3Kac14) (Ferguson et al., 2013).

### Structure-Based Design

The rationally structure-based design of protein-protein interaction abrogators has been considered challenging due to the large and shallow interfaces of the interaction domains with the lack of endogenous ligands as a starting point to be chemically mimicked. However, structural studies (alanine scanning mutagenesis and X-ray crystallography) identifying peptide fragments and amino acid residues critical for the protein-protein interactions, or the so-called ‘‘hot-spots’’, provided essential structural information and a solid basis for the rational design of protein-protein interaction abrogators (Ivanov et al., 2013; Lu et al., 2020). Two structure-based design strategies are currently implemented. The first one is the hot spot-based design of small molecules and peptide inhibitors (Guo et al., 2014). The second is the peptidomimetic design of peptide-like molecules to mimic the 3D structures of the original protein-protein interaction interfaces for the sake of competitive inhibition and subsequent disruption (Wang et al., 2021). Structure-based design strategies were previously applied in the successful identification of protein-protein interaction inhibitors, for instance, small molecule inhibitors for the interaction of Myeloid Leukemia 1 (Mcl-1) with BH3-containg peptides (Kotschy et al., 2016), small molecule inhibitor (Chessari et al., 2021) and p53 α-helical peptide mimetic inhibitor (Chen et al., 2005) of p53 interaction with Mdm2.

## Compounds That Abrogate the Interaction Between Mortalin and p53

Due to the well-known consequences of the cytoplasmic mortalin-p53 interaction, for instance, the contribution to the malignant transformation of NIH/3T3 cells (Wadhwa et al., 1999) and the life span extension of the human diploid fibroblast MRC-5 leading to a non-permanent escape from cellular senescence (Kaula et al., 2000), abrogation of this interaction as an anti-cancer therapeutic concept emerged (Wadhwa et al., 2000; Wadhwa et al., 2002). Major compounds that have been reported to abrogate mortalin-p53 interaction are listed in Table 1 and explained below.

**TABLE 1:** | Compounds that have been reported to abrogate the interaction between mortalin and p53.

Molecule	Type	Mode of abrogation and validation assays	Structure^a^	References
MKT-077			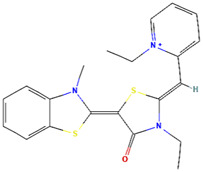	(Tikoo et al., 2000; Wadhwa et al., 2000)
SHetA2			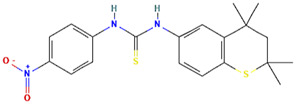	Liu et al. (2004)
Az-TPP-O3	Synthetic Compounds	Direct abrogation validated *in vitro* by indirect co-immunoprecipitation (Co-IP)	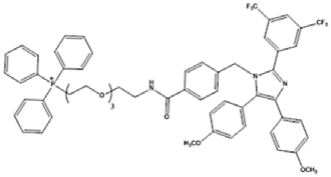	(DeRan and Wagner, 2018; Park et al., 2018)
Mortaparib^Plus^			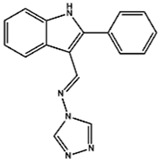	(Elwakeel et al., 2021; Sari et al., 2021)
Withanone			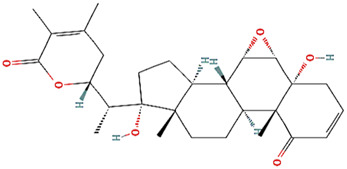	Grover et al. (2012)
Caffeic Acid Phenethyl Ester (CAPE)	Naturally occurring compounds	Direct abrogation validated by *in silico* analyses and indirect co-immunoprecipitation (Co-IP)	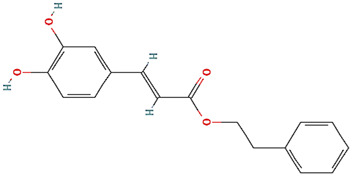	(Wadhwa et al., 2016; Sari et al., 2020)
Artepillin C			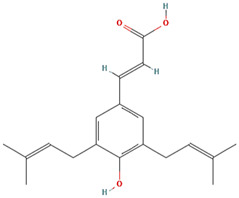	Bhargava et al. (2018)
Fucoxanthin			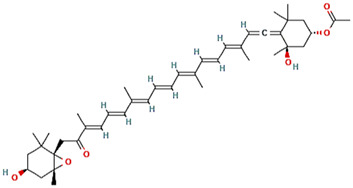	(Wang et al., 2014; Garg et al., 2019)
Solasonine	Naturally occurring compounds	Direct abrogation validated by *in silico* analyses	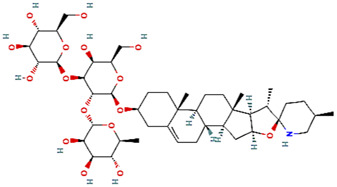	Pham et al. (2019)
Embelin			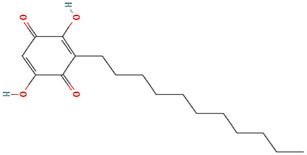	Nigam et al. (2015)
Campesterol			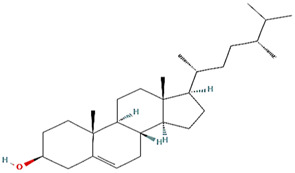	Hartati and Djauhari, (2020)
Veratridine	Naturally occurring compound	Indirect abrogation	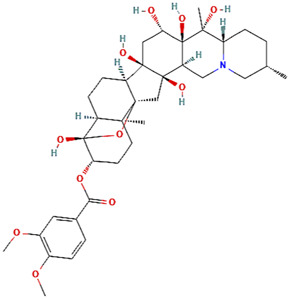	(Sane et al., 2014; Abdullah et al., 2015)

a All structures are downloaded from PubChem database (except for Az-TPP-O3 and Mortaparib^Plus^ are drawn by ChemSketch).

### MKT-077

MKT-077, formerly known as FJ776, (1-Ethyl-2-{[3-ethyl-5- (3-methylbenzothiazolin-2 yliden)]-4-oxothiazolidin-2-ylidenemethyl}pyridium chloride) is a water soluble delocalized lipophilic cation (DLC) dye. It was firstly synthesized at Fuji Photo Film Co. (Ashigara, Kanagawa, Japan) (Chiba et al., 1998). Due to the selective accumulation in cancer cells’ mitochondria (characterized by a higher membrane potential), MKT-077 had been investigated in several preclinical studies as an anti-cancer molecule (Modica-Napolitano et al., 1996; Chiba et al., 1998; Petit et al., 1999; Propper et al., 1999; Tatsuta et al., 1999). As mortalin has been previously identified and affinity purified as an MKT-077 target protein in v-Ha-*ras*-transformed NIH/3T3 fibroblasts (Tikoo et al., 2000), Renu Wadhwa and colleagues conducted investigations to define the binding region of mortalin to MKT-077 (Wadhwa et al., 2000). Interestingly, it was found to be within the amino acid residues 252–310 [a motif overlapped with the p53’s binding region (amino acid residues 253–282) (Kaul et al., 2001)]. Accordingly, and as expected, mortalin was not co-immunoprecipitated with p53 from the lysates of MKT-077-treated MCF-7 breast adenocarcinoma cells, EJ Human endometrial adenocarcinoma cells, and COS7 kidney fibroblasts. Hence, it was concluded that MKT-077 dissociated mortalin from p53 and this abrogation was accompanied by an activation of the p53 transcriptional activity as depicted by the upregulation in the p21^CIP1/WAF1^ protein levels and the enhanced transcription from a p53-responsive promoter (Wadhwa et al., 2000). Although MKT-077 did not pass a human phase I clinical trial against advanced solid tumors due to excessive renal toxicity (Propper et al., 1999), this compound is still used as a starting point for designing derivatives with improved potency and metabolic stability to inhibit mortalin and other Hsp70-family members (Miyata et al., 2013; Shao et al., 2018).

### SHetA2

Sulphur Het A2 (SHetA2) {[ (4-nitrophenyl)amino] [2,2,4,4-tetramethylthiochroman-6-yl)amino]methanethione], NSC726189} is a synthetic small Flexible Heteroarotinoid (Flex-Het) lead compound emerged from a series of structure activity relationship (SAR) studies of retinoic acid receptor-active heteroarotinoids (Benbrook et al., 1997; Zacheis et al., 1999; Chun et al., 2003; Liu et al., 2004). Based on previous preclinical studies that have shown no evident mutagenic, carcinogenic, teratogenic, or toxic effects for SHetA2 (Benbrook et al., 2014; Ramraj et al., 2020), and based on its promising anti-cancer (Nammalwar et al., 2013) and chemo-preventive effects (Benbrook et al., 2013; Kabirov et al., 2013; Benbrook et al., 2018), currently, a first-in-human Phase-I clinical trial is being conducted for SHetA2 capsules in patients with advanced or recurrent ovarian, cervical, and endometrial cancer (ClinicalTrials.gov Identifier: NCT04928508, accessed 6^th^ February, 2022). Previously, to identify the SHetA2-binding proteins that may be responsible for its anti-ovarian cancer effects (Liu et al., 2004), Doris Benbrook and colleagues synthesized an SHetA2 metabolite to allow the attachment of a linker molecule required for the affinity chromatography experimental concept of identifying small molecule target proteins. Then, after extensive synthetic chemical reactions, they attached the SHetA2 metabolite to a magnetic microsphere through a linker molecule as a physical separation between SHetA2 and the microsphere scaffold. After that, through conducting affinity chromatography analyses, SHetA2-conjugated microspheres suspensions were used to identify the SHetA2 protein targets from the protein extracts of A2780 ovarian cancer cells. After subjecting the excised 75 kDa SDS-PAGE band that was differentially present in the lanes corresponding to the SHetA2-conjugated and unconjugated microsphere eluents to mass spectrometric analyses, mortalin was identified to be specifically bound by SHetA2. Finally, through the implementation of the indirect co-immunoprecipitation analyses, p53 was not co-immunoprecipitated with mortalin from SHetA2-treated A2780 ovarian cancer cell lysates confirming the ability of SHetA2 to disrupt the interaction of mortalin and p53.

### Az-TPP-O3

Az-TPP-O3 is apoptozole (4-[[2-[3,5-bis (trifluoromethyl)phenyl]-4,5-bis (4-methoxyphenyl)imidazol-1-yl]methyl]benzamide) conjugated with the well-known mitochondria-targeting motif triphenylphosphine (TPP). Apoptozole is a synthetic small molecule that has been previously reported to inhibit the ATPase activity of Hsp70 by binding to its ATPase domain (without affecting Hsp40, Hsp60, and Hsp90) inducing caspase-dependent apoptotic cancer cell death *in vitro* and abrogating tumor growth *in vivo* (Ko et al., 2015; Park et al., 2021). Sang-Hyun Park and colleagues had taken the advantage of the differential subcellular localization of Hsp70 family members to provide a more detailed mechanistic characterization of apoptozole as a selective inhibitor of Hsp70 (DeRan and Wagner, 2018; Park et al., 2018). Firstly, upon treatment of cancer cells with the unmodified apoptozole, it accumulated in the lysosomes to specifically inhibit lysosomal Hsp70 (without affecting the activity of cytosolic Hsp70) leading the induction of lysosomal membrane permeabilization (LMP) and caspase-mediated apoptosis. Secondly, to target the mitochondrial Hsp70 mtHsp70 (mortalin), apoptozole had been conjugated with a mitochondrial-targeting triphenylphosphine (TPP) moiety to create Az-TPP-O3. Such a modification resulted in more potent induction of apoptosis than that induced by the unmodified apoptozole with similar inhibitory effects on the ATPase domain of Hsp70. Mechanistically, Az-TPP-O3 contributed to the abrogation of mortalin-associated protein-protein interactions. Through the implementation of the indirect co-immunoprecipitation analyses, Sang-Hyun Park and colleagues had reported a dose-dependent decrease in mortalin fractions that were co-immunoprecipitated with equal amounts of p53 from Az-TPP-O3-treated HeLa cancer cell lysates. Accordingly, it has been concluded that Az-TPP-O3 abrogated the interaction of mortalin with p53 leading to mitochondrial outer membrane permeabilization (MOMP), release of cytochrome C, and apoptosis.

### Mortaparib^Plus^

Mortaparib^Plus^ (4-[ (1E)-2- (2-phenylindol-3-yl)-1-azavinyl]-1,2,4-triazole) is a novel synthetic small molecule that was isolated from a chemical library (12,000 molecules) after a high-throughput screening (HTS) assay based on two bioimage redistributive readouts [the shift of mortalin staining pattern from perinuclear (typical for cancer cells) to pan-cytoplasmic (typical for normal cells) and the stabilization, accumulation and nuclear enrichment of p53] (Putri et al., 2019). Such readouts (the differential staining of mortalin and the change of p53 localization) were previously reported as criteria for the selection of an anti-cancer molecule with the potentiality to abrogate mortalin-p53 interaction (Gao et al., 2012; Putri et al., 2019). Then, to validate such a potentiality *in silico*, molecular docking analyses revealed that Mortaparib^Plus^ could bind to mortalin at the interface of p53 binding site and molecular-dynamics simulation of 100ns in the explicit water-model system revealed that Mortaparib^Plus^-mortalin complex was quite stable (Sari et al., 2021). Accordingly, with the aim to validate these *in silico* results, indirect co-immunoprecipitation analyses were performed. Equal immunoprecipitated mortalin complexes showed a decrease in p53 fractions in the treated HCT116, DLD-1, MCF7, and T47D cells as compared to their respective untreated controls confirming the capability of Mortaparib^Plus^ to abrogate the interaction of mortalin and p53 regardless of the p53 status (wild type or point mutant) (Elwakeel et al., 2021; Sari et al., 2021).

### Phytochemicals and Other Naturally Occurring Compounds

Previously, based on empirical observations rather than high throughput and/or virtual screening campaigns, multiple naturally occurring molecules have been reported to abrogate the interaction between mortalin and p53. For instance, mainly based on *in silico* analyses (docking studies and/or molecular dynamic simulations of the docked complexes), Artepillin C (Bhargava et al., 2018), Fucoxanthin (Wang et al., 2014; Garg et al., 2019), Solasonine (Pham et al., 2019), Embelin (Nigam et al., 2015), and Campesterol (Hartati and Djauhari, 2020) have been shown as mortalin-p53 interaction disruptors. At an *in vitro* level (with the exception of Campesterol), these phytochemical compounds have been reported to stabilize and accumulate p53 in the nuclei of different cancer cell lines, possibly through the disruption of mortalin-p53 complexes (Wang et al., 2014; Nigam et al., 2015; Bhargava et al., 2018; Garg et al., 2019; Pham et al., 2019). However, common biochemical methodologies, for instance, affinity chromatography approaches (pull-down and co-immunoprecipitation assays) and fluorescence or bioluminescence resonance energy transfer approaches (FRET or BRET), have not been implemented to mechanistically confirm the potentiality of these molecules as mortalin-p53 interaction inhibitors (Zhou et al., 2018). Additionally, based on *in silico* analyses that were further validated by *in vitro* co-immunoprecipitation assays, Caffeic Acid Phenethyl Ester (CAPE) (Wadhwa et al., 2016; Sari et al., 2020) and Withanone (Grover et al., 2012) have been previously reported as naturally occurring molecules with the capability to abrogate mortalin-p53’s interaction. Yet another phytochemical molecule, Veratridine (VTD), has been reported to enhance the transactivation of Ubiquitin-like (UBX)-domain-containing protein (UBXN2A) (Abdullah et al., 2015). UBXN2A has been previously reported to bind to mortalin in a binding site overlapped with a p53’s binding motif, and consequently, competitively inhibit the binding between mortalin and p53 (Sane et al., 2014). Hence, Veratridine (VTD) could indirectly dissociate mortalin-p53’s interaction.

## Targeting Mortalin-p53 Interaction: Where do We Stand?

Generally, an intimate knowledge of the interaction interface between two proteins is considered a prerequisite to inform either a structure-based, *in silico*-based or fragment-based drug discovery efforts targeting their interaction. This could be provided by the detailed atomic structure of the interaction interface by X-ray crystallography or protein-based nuclear magnetic resonance (NMR) spectroscopy (Scott et al., 2016). For instance, elucidation of the MDM2 crystal structure in complex with the N-terminal peptide of p53 (Kussie et al., 1996) has previously guided the rational-based synthesis of the terphenyl scaffold molecules mimicking the N-terminal α-helix of p53 and blocking the p53/MDM2 interaction (Chen et al., 2005). Additionally, the NMR-based elucidation of the 3D structure of BH3 peptides from Bak and Bad bound to Bcl-xL (Sattler et al., 1997; Petros et al., 2000) has guided the fragment-based identification of a potent inhibitor for Bcl-xL (Petros et al., 2006). Furthermore, the virtual screening-based identification of the p53/MDM2 interaction inhibitor (NSC 333003) (Lawrence et al., 2009) has previously relied upon the X-ray crystal structure of a truncated human MDM2 in complex with an optimized p53 peptide (PDB ID: 1T4F). Recently, the classification of the protein-protein interaction interfaces based upon the complexity of their binding epitopes has been discussed and the druggability of these interface classes has been argued. For instance, the interaction between a pair of globular proteins, for example interleukin-2 (IL-2) and its receptor (IL-2R), and the interaction between two peptidic regions, for example c-MYC and MYC-associated factor X (MAX), are challenging drug targets. However, other protein-protein interaction interfaces have been proposed to be more druggable, for instance, the interaction of one partner protein through a single peptidic region with another globular partner protein (Scott et al., 2016). This peptidic region usually acquires one or more secondary structural elements upon binding to the globular interacting protein and these elements may or may not be present in the unbound state. For this more druggable class, and with the emergence of the ‘‘hot-spot’’ concept (certain hot spot residues or regions are largely responsible for driving the binding affinity of a pair of proteins), lead discovery efforts led to the identification of small molecules that typically mimic the interactions made by the peptide and place groups into the hot-spot pockets on globular proteins, for instance, the interaction of RAD51 with breast cancer type 2 susceptibility protein (BRCA2) (Scott et al., 2015; Scott et al., 2021).

In the case of mortalin and p53, to date, there is no access to the full 3D structure of their complete interaction or the interaction of their truncated or modified versions to well-inform drug discovery efforts. Furthermore, different independent studies controversially reported the binding domains of mortalin and p53 (Kaul et al., 2001; Wadhwa et al., 2002; Kaul et al., 2005; Iosefson and Azem, 2010; Gabizon et al., 2012). Accordingly, without ample 3D structural biology studies, it is hard to predict if mortalin-p53 interaction is a formidable or a truly druggable target. In fact, high throughput screening (HTS) could still be implemented to identify a ‘‘hit’’ small molecule inhibitor of mortalin-p53 interaction because, theoretically, HTS does not require the elucidation of the full 3D structure of the interaction if a suitable validation scheme is available (Chang et al., 2021; Elwakeel et al., 2021; Sari et al., 2021). However, there are multiple limitations for such HTS-based attempts. For instance, the limited chemical space of the conventional compound libraries that have been previously constructed for classical drug targets (enzymes or receptors) urged the utilization of the newly established and innovative compound libraries (MCR-inspired, DOS-inspired, and Cascade reaction-inspired libraries). Furthermore, HTS often results in false positive hits or artefacts (Baell and Walters, 2014). Hence, positive hits need to be further and more comprehensively validated to rule out any false positive artefacts. Post-screening hit validation includes (i) the characterization of the small molecule–target proteins interaction by the determination of the binding kinetics [using Surface plasmon resonance (SPR), Bio-layer interferometry, Isothermal titration calorimetry, or Microscale thermophoresis] and the complex structure [X-ray crystallography or Nuclear magnetic resonance (NMR)] (Choi and Choi, 2017); and (ii) cell-based *in vitro* validation through pull-down, co-immunoprecipitation (Co-IP), or luciferase reporter assays. In the case of Mortaparib^Plus^, a small molecule identified after a Bio-Image Redistribution-based HTS step of a conventional compound library (only 12,000 synthetic and natural compounds), only co-immunoprecipitation (Co-IP) assays from cell lysates were performed as an *in vitro* post-screening validation approach, and hence, further studies to investigate the binding kinetics and Mortaparib^Plus^-p53 or Mortaparib^Plus^-mortalin complex structures are strongly recommended as a future direction for validation. Furthermore, regardless of the encouraging variety of molecular sizes and chemical structures of the secondary metabolites from natural sources that motivated the previous application of high throughput screening strategies to discover protein-protein interaction inhibitors from chemical libraries of naturally occurring molecules (Lepourcelet et al., 2004; Hashimoto et al., 2009; Ferrari et al., 2013), all the previously-reported natural-based inhibitors of mortalin-p53 interaction were identified based on empirical observations or trial-and-error approaches rather than high throughput screening campaigns (Grover et al., 2012; Wang et al., 2014; Nigam et al., 2015; Wadhwa et al., 2016; Bhargava et al., 2018; Garg et al., 2019; Pham et al., 2019; Hartati and Djauhari, 2020; Sari et al., 2020). For instance, regardless of the controversy of the interacting domains of mortalin and p53 (Kaul et al., 2001; Wadhwa et al., 2002; Kaul et al., 2005; Iosefson and Azem, 2010; Gabizon et al., 2012), Artepillin C (Bhargava et al., 2018), Fucoxanthin (Garg et al., 2019), and Embelin (Nigam et al., 2015) were biasedly identified based on their docking into the ATPase domain of mortalin (PDB ID: 4KBO) and tetramerization domain of p53 (PDB: 1OLG) neglecting other reported mortalin-p53 interaction domains, namely, C-terminal domain of p53 and peptide-binding domain of mortalin. Accordingly, common biophysical and biochemical methodologies that could sufficiently confirm the potentiality of those natural-based inhibitors are strongly recommended as a next step. Additionally, for the application of HTS to screen chemical libraries of naturally occurring compounds, it is noteworthy to mention that, from a structure-activity relationship (SAR) perspective, positive hits from these libraries are challenging to optimize in the post-HTS stages (Scott et al., 2016).
